# Cardiovascular Changes in Pregnant Women After Mild COVID-19 Infection: An Examination of Endothelial and Autonomic Functions

**DOI:** 10.7759/cureus.117799

**Published:** 2026-10-12

**Authors:** Hande Seymen, Ihsan Safak, Muhammed Emre Gülesir, Asli Kurtar Mansiroglu

**Affiliations:** 1 Department of Cardiology, Sultan 1st Murad State Hospital, Edirne, TUR; 2 Department of Gynecology, Bolu Abant Izzet Baysal University, Bolu, TUR; 3 Department of Cardiology, Izzet Baysal State Hospital, Bolu, TUR; 4 Department of Cardiology, Kirklareli University Faculty of Medicine, Kirklareli, TUR

**Keywords:** covid-19, echocardiography, epicardial adipose tissue, flow-mediated dilation, heart rate, platelet-to-lymphocyte ratio, pregnancy, vasodilation

## Abstract

Introduction: Pregnant women constitute a special group regarding the cardiovascular effects of coronavirus disease 2019 (COVID-19). We employed transthoracic echocardiography, 24-hour rhythm Holter monitoring, and flow-mediated dilation (FMD) to evaluate the cardiac implications of COVID-19 in pregnant women diagnosed within the past six months.

Methods: This study involved 80 pregnant women, 36 of whom were diagnosed with COVID-19 in the last six months of their pregnancy, while 44 served as healthy control subjects. The findings of transthoracic echocardiography, FMD, and 24-hour rhythm Holter monitoring were analyzed in all participants. All results were deemed statistically significant at a p-value threshold of less than 0.05.

Results: Echocardiography, FMD, and Holter monitoring of the COVID-19 group were performed 43 days (range: 32-174) after diagnosis. FMD values were lower in the COVID-19 group (7.90 (3.45-14.09)% vs. 14.30 (10.32-22.56)%, p=0.005). Epicardial adipose tissue (EAT) was thicker in the COVID-19 group (p=0.021). Systolic pulmonary artery pressure (sPAP) was lower in the COVID-19 group (p=0.013). The platelet-to-lymphocyte ratio (PLR) was higher in patients with a history of COVID-19 (157.36 (129.10-195.39) vs. 118.90 (101.10-152.29), p<0.001). Other echocardiographic and Holter parameters showed no differences. Adjusting for pregnancy week in a multivariable model attenuated the association between COVID-19 history and FMD (p=0.122). Gestational age acts as a confounder.

Conclusions: Mild COVID-19 during pregnancy is associated with lower unadjusted FMD and higher PLR in the recovery phase. Gestational age is a major confounder. Future studies require strict age matching.

## Introduction

A novel coronavirus, initially identified in Wuhan, China, and rapidly disseminated globally, was named coronavirus disease 2019 (COVID-19) and officially classified as a pandemic by the World Health Organization in March 2020 [1].

COVID-19, a single-stranded ribonucleic acid (RNA) virus, affects a wide range of patients, from asymptomatic to fatal disease. Although COVID-19 primarily affects the respiratory and digestive systems, it can also affect endothelial cells of arteries and vessels, arterial smooth muscle, and immune cells via angiotensin-converting enzyme 2 receptors [2].

Studies examining the course of COVID-19 in pregnant women have shown that the need for intensive care unit (ICU) admission and mechanical ventilation is higher than in nonpregnant women [3]. Another retrospective study demonstrated a lower need for hospital admission [4]. In another study, no statistically significant difference was found in ICU admission rates between the two groups [5].

The most common symptoms of COVID-19 in pregnant women include fever, cough, dyspnea, diarrhea, and malaise, similar to those in nonpregnant women [6,7].

According to a cohort published by the World Health Organization, only 8% of pregnant women with COVID-19 are severely ill, and 1% are critically ill [8]. In our study, only pregnant patients with mild illness were included; thus, the aim was to evaluate the cardiac effects of mild illness, which constitutes the majority of COVID-19 cases in pregnant women.

Limited data exist in the literature on the cardiovascular effects of COVID-19 in pregnant women. Moreover, our study is the first to evaluate endothelial function with flow-mediated dilation (FMD) and examine heart rate variability (HRV) with rhythm Holter monitoring.

This was a comparative cross-sectional study. We evaluated pregnant women with a recent history of mild COVID-19 and matched controls. We used transthoracic echocardiography, FMD, and Holter monitoring. The primary outcome was FMD. We assessed endothelial and autonomic functions.

## Materials and methods

This single-center study was conducted between May 2021 and October 2021. The study included 36 pregnant women who were diagnosed with COVID-19 during their pregnancy within the past six months and did not require hospitalization, as well as 44 healthy pregnant control subjects. A real-time reverse transcriptase polymerase chain reaction (RT-PCR) assay was used for diagnosis. All patients with COVID-19 included in the study were in the early recovery phase.

The exclusion criteria were as follows: left ventricular ejection fraction (EF) <50%, history of coronary artery disease, moderate and advanced valvular disease, congenital heart disease, atrioventricular conduction defects, pacemaker use, antiarrhythmic drug use, chronic lung disease, moderate and advanced kidney and liver disease, thyroid disease, electrolyte imbalance, systemic inflammatory disease, poor echocardiographic image quality, and poor-quality rhythm Holter recordings. The Local Ethics Committee approved the study protocol, and written informed consent was obtained from each participant prior to participation.

The clinical classification of the study participants was conducted in accordance with the Diagnosis and Treatment Plan for COVID-19 established by the National Health Commission (7th edition). According to this classification, mild disease is characterized by mild clinical symptoms without pneumonia on radiological imaging [9].

Although the accepted cumulative radiation dose in pregnant women is 5 rad and 10 chest CT slices lead to <0.1 rad exposure [10,11], CT was not indicated, and the total severity score (TSS) classification was not performed due to the mild clinical condition of the patients included in our study [12].

We obtained laboratory data for the COVID-19 group at hospital admission and collected control group data at study enrollment. We calculated systemic inflammatory indices from routine complete blood counts. These included the platelet-to-lymphocyte ratio (PLR) and neutrophil-to-lymphocyte ratio (NLR) [13]. The systemic immune-inflammation index (SII) was calculated as (platelets × neutrophils)/lymphocytes [14].

All patients and controls were evaluated with transthoracic echocardiography, FMD, and 24-hour rhythm Holter monitoring, in addition to laboratory parameters. The participants included in the study were not followed long-term (Figure 1).

**Figure 1:**
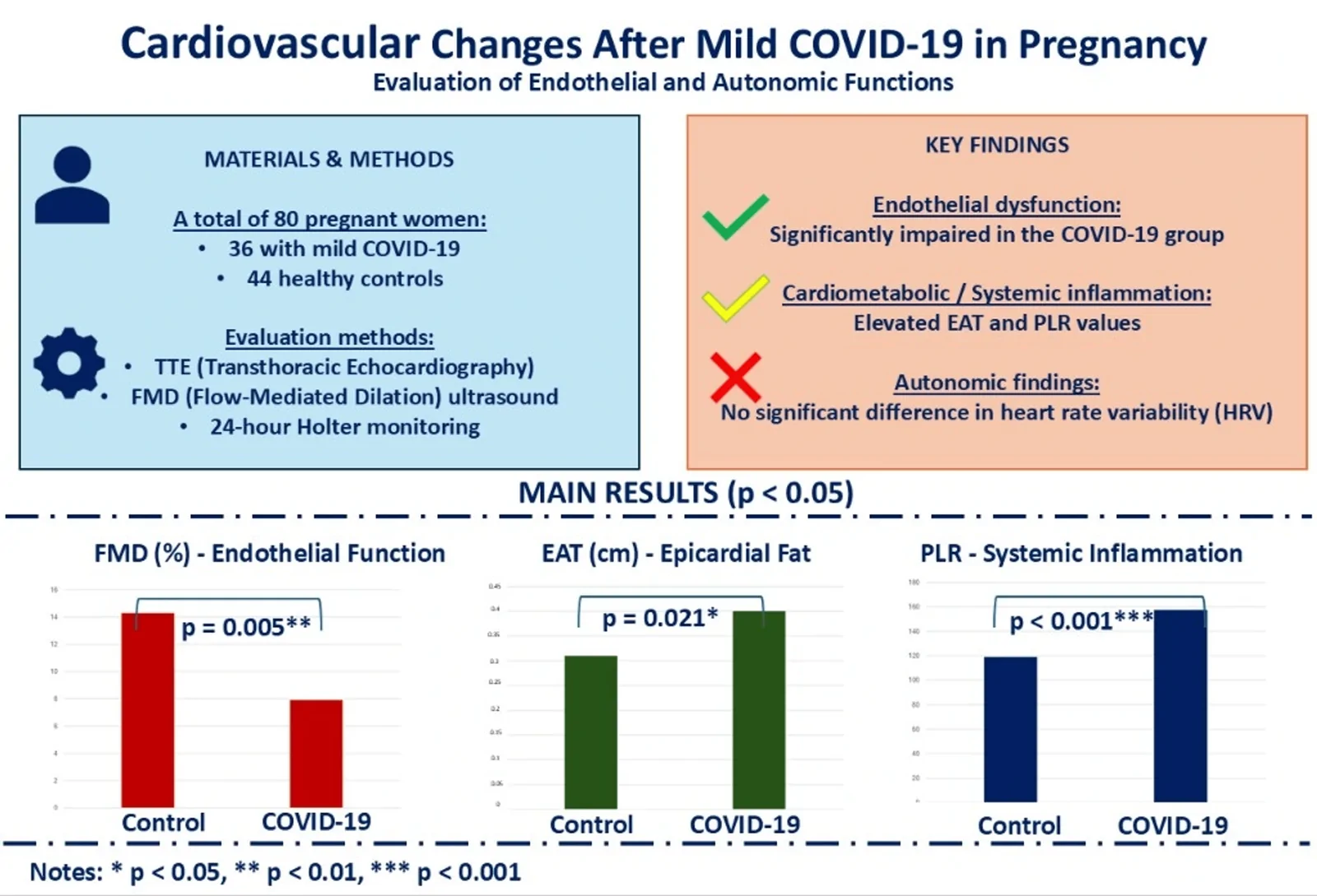
Study design, key findings, and results This figure was created using Microsoft PowerPoint (Microsoft Corporation, Redmond, WA, USA). TTE, transthoracic echocardiography; FMD, flow-mediated dilation; HRV, heart rate variability; EAT, epicardial adipose tissue; PLR, platelet-to-lymphocyte ratio

Echocardiography

Echocardiographic assessments were conducted by a single-blinded cardiologist with the patient positioned in the left lateral position, using a 4-MHz transducer from the Vivid S6 (GE Vingmed, N-3191 Horten, Norway) with continuous ECG monitoring. Body mass index (BMI) was calculated as weight in kilograms divided by height in meters squared.

A biplane-modified Simpson method was used to measure left ventricular EF. Two-dimensional and pulsed Doppler measurements were conducted in accordance with the standards established by the European Association of Cardiovascular Imaging [15].

The systolic (AoSD) and diastolic (AoDD) measurements of the ascending aorta were obtained by M-mode echocardiography using the average of three consecutive measurements approximately 3 cm above the aortic valve level in the parasternal long-axis view. Aortic diameter change (ADC), aortic strain (AS), and aortic stiffness index (ASI) were calculated with the following formulas [16]: AS=ADC/AoDD; ADC=AoSD-AoDD; ASI=ln(SBP/DBP)/((ADC)/AoDD); aortic distensibility (cm² dyn⁻¹×10⁻³)=2 AS/PP (pulse pressure).

Epicardial adipose tissue (EAT) was measured from the parasternal long-axis view, with the echo-free space between the myocardial outer wall adjacent to the right ventricular free wall and the visceral pericardium positioned perpendicular to the aortic annulus at the end of systole [17].

Ultrasonography

Ultrasonographic measurements were made using a 7.5-MHz linear array transducer (GE Healthcare, M4S-RS, Tokyo, Hino-Shi, Japan) in a dim, quiet, well-ventilated environment at a room temperature of 22-25°C after the patient rested for at least 15 minutes. In addition, participants were asked to fast and stay away from cigarettes, alcohol, caffeine, and exercise for eight hours before the test. Measurements were made by marking the skin with a pencil to study the brachial artery at the same level in the antecubital fossa. We placed a blood pressure cuff on the forearm. We measured the baseline brachial artery diameter. We inflated the cuff to 50 mmHg above systolic pressure for 5 minutes. After deflation, we monitored the artery continuously. We recorded the maximum diameter during the first minute. FMD was calculated with the formula below: FMD=100×(maximum diameter during the first minute - baseline diameter)/baseline diameter [18].

Rhythm Holter evaluation

Twenty-four-hour ambulatory electrocardiographic Holter monitoring was conducted using a Cardioline S.P.A Cube 3-channel recorder (Cardioline S.p.A., Trento, Italy). The recordings were analyzed using the Cardioline Analysis System. All recordings were visually assessed and manually reviewed by an experienced cardiologist to confirm beat classification. The minimum acceptable recording duration was 18 hours. We excluded ectopic beats and artifacts from the analysis. The following parameters were used to evaluate HRV in the time domain: mean of the SD in all 5-min intervals (HRVM (milliseconds)), SD in all 5-min intervals (SDHRV (milliseconds)), SD of the mean of all normal-to-normal intervals in all consecutive 5-min segments of the entire recording (SDANN (milliseconds)), SD of all normal-to-normal intervals (SDNN (milliseconds)), and the mean square root of differences between adjacent normal-to-normal intervals (rMSSD (milliseconds)).

Statistical analysis

A priori power analysis was conducted using G*Power version 3.1 software (Heinrich Heine University Düsseldorf, Düsseldorf, Germany) to justify the sample size. To detect a medium-to-large effect size (Cohen's d=0.65) in FMD and related cardiovascular parameter differences between the two independent groups with an alpha error probability of 0.05 and a statistical power (1-β) of 0.80, a minimum total sample size of 76 patients was required. Accounting for potential attrition or missing data, we enrolled a total of 80 participants. All statistical analyses were conducted using SPSS 18.0 Statistical Package Software for Windows (SPSS Inc., Chicago, IL, USA). We handled missing data using pairwise deletion. This approach maximizes the use of available data. Analysis-specific denominators are reported in the respective tables. The normality of the variables was assessed using the Kolmogorov-Smirnov test. Continuous variables with a normal distribution were described using mean and SD; continuous variables without a normal distribution were described using median and interquartile range. Data are presented as numbers or percentages for qualitative variables. To analyze differences between independent groups, we used a two-tailed Student's t-test for normally distributed quantitative variables, Mann-Whitney U-test for variables without a normal distribution, and Pearson chi-square and Fisher's exact tests for qualitative variables. A p-value of less than 0.05 indicated statistical significance. Correlation analysis between continuous variables was performed using Spearman's rank correlation coefficient. Receiver operating characteristic (ROC) curve analysis was used to assess the predictive capabilities of continuous variables.

## Results

Demographic details

Most baseline clinical features, including smoking status (p=0.974), were similar between the groups. However, the COVID-19 group had a higher mean pregnancy week (p=0.050). Additionally, systolic blood pressure was significantly lower in the COVID-19 group (104.72±10.82 vs. 110.80±13.38 mmHg, p=0.031) (Table 1).

**Table 1:** Demographic and baseline clinical characteristics of the study population Data are expressed as mean±SD for normally distributed variables (*), as median (25th-75th percentiles) for non-normally distributed variables (**), and as number (percentage) for categorical variables (***). *Independent samples t-test, **Mann-Whitney U test, **Fisher's exact test/Pearson chi-square. BMI, body mass index; SBP, systolic blood pressure; DBP, diastolic blood pressure; CAD, coronary artery disease

Variables	N (control/COVID-19)	Control group	COVID-19 group	p-value
Pregnancy week	44/36	24.77±7.82	28.39±8.41	0.050*
Number of pregnancies	44/36	1.50 (1.00-2.00)	1.00 (1.00-2.00)	0.422**
Number of abortus	44/36	0.00 (0.00-0.00)	0.00 (0.00-0.00)	0.300**
Number of born alive	44/36	0.00 (0.00-1.00)	0.00 (0.00-0.00)	0.460**
Age (years)	44/36	29.50±6.04	31.58±5.36	0.111*
Height (cm)	44/36	160.00±5.88	161.33±6.23	0.329*
Weight (kg)	44/36	74.72±16.82	74.22±16.38	0.895*
BMI (kg/m²)	44/36	28.90±6.17	28.56±6.55	0.814*
SBP (mmHg)	44/36	110.80±13.38	104.72±10.82	0.031*
DBP (mmHg)	44/36	71.70±10.94	68.19±9.27	0.131*
Pulse pressure (mmHg)	44/36	39.09±8.23	36.53±6.74	0.137*
Hypertension, n (%)	44/36	1 (2.3%)	0 (0.0%)	1.000***
Diabetes mellitus, n (%)	44/36	1 (2.3%)	3 (8.3%)	0.322***
Hyperlipidemia, n (%)	44/36	0 (0.0%)	0 (0.0%)	-
Family history of CAD, n (%)	44/36	12 (27.3%)	9 (25.0%)	0.818***
Smoking, n (%)	44/36	6 (13.6%)	5 (13.9%)	0.974***

Laboratory findings

White blood cell and neutrophil counts were lower in the COVID-19 group (p=0.023 and p=0.005). Platelet counts and alanine aminotransferase (ALT) levels were higher (p=0.038 and p=0.011). PLR was higher in the COVID-19 group (p<0.001) (Table 2, Figure 2).

**Table 2:** Laboratory variables of the study population Data are expressed as mean±SD for normally distributed variables (*), and as median (25th-75th percentiles) for non-normally distributed variables (**). *Independent samples t-test; **Mann-Whitney U test. WBC, white blood cell; AST, aspartate transaminase; ALT, alanine aminotransferase; CRP, C-reactive protein; NLR, neutrophil-to-lymphocyte ratio; PLR, platelet-to-lymphocyte ratio; SII, systemic immune-inflammation index

Variables	N (control/COVID-19)	Control group	COVID-19 group	p-value
Uric acid (mg/dL)	12/11	3.66±1.25	3.75±0.89	0.833*
Urea (mg/dL)	44/36	16.00 (13.00-20.00)	16.00 (13.00-17.00)	0.701**
Creatinine (mg/dL)	44/36	0.60±0.07	0.61±0.07	0.501*
Fasting plasma glucose (mg/dL)	44/36	81.50 (73.00-93.50)	84.00 (76.25-111.00)	0.137**
WBC (K/uL)	44/36	9.75±2.32	8.51±2.45	0.023*
Hemoglobin (g/dL)	44/36	11.81±1.73	12.07±1.32	0.459*
Hematocrit (%)	44/36	35.45 (32.68-38.90)	36.05 (34.00-38.70)	0.753**
Platelet counts (K/uL)	44/36	209.00 (189.75-243.25)	261.00 (189.75-301.25)	0.038**
Lymphocyte	44/36	1.79±0.48	1.61±0.70	0.179*
Monocyte	44/36	0.68 (0.59-0.89)	0.64 (0.50-0.85)	0.341**
Neutrophil	44/36	7.11 (6.51-8.31)	5.43 (4.78-7.36)	0.005**
Basophil	44/36	0.03 (0.02-0.05)	0.03 (0.01-0.05)	0.242**
Eosinophil	44/36	0.06 (0.04-0.12)	0.05 (0.02-0.10)	0.265**
AST (U/L)	44/36	17.50 (14.00-21.75)	18.50 (14.00-22.00)	0.981**
ALT (U/L)	44/36	12.00 (8.25-14.00)	14.00 (11.00-23.50)	0.011**
D-dimer (mg/L)	26/18	1.21 (1.04-2.09)	0.96 (0.63-1.35)	0.083**

**Figure 2:**
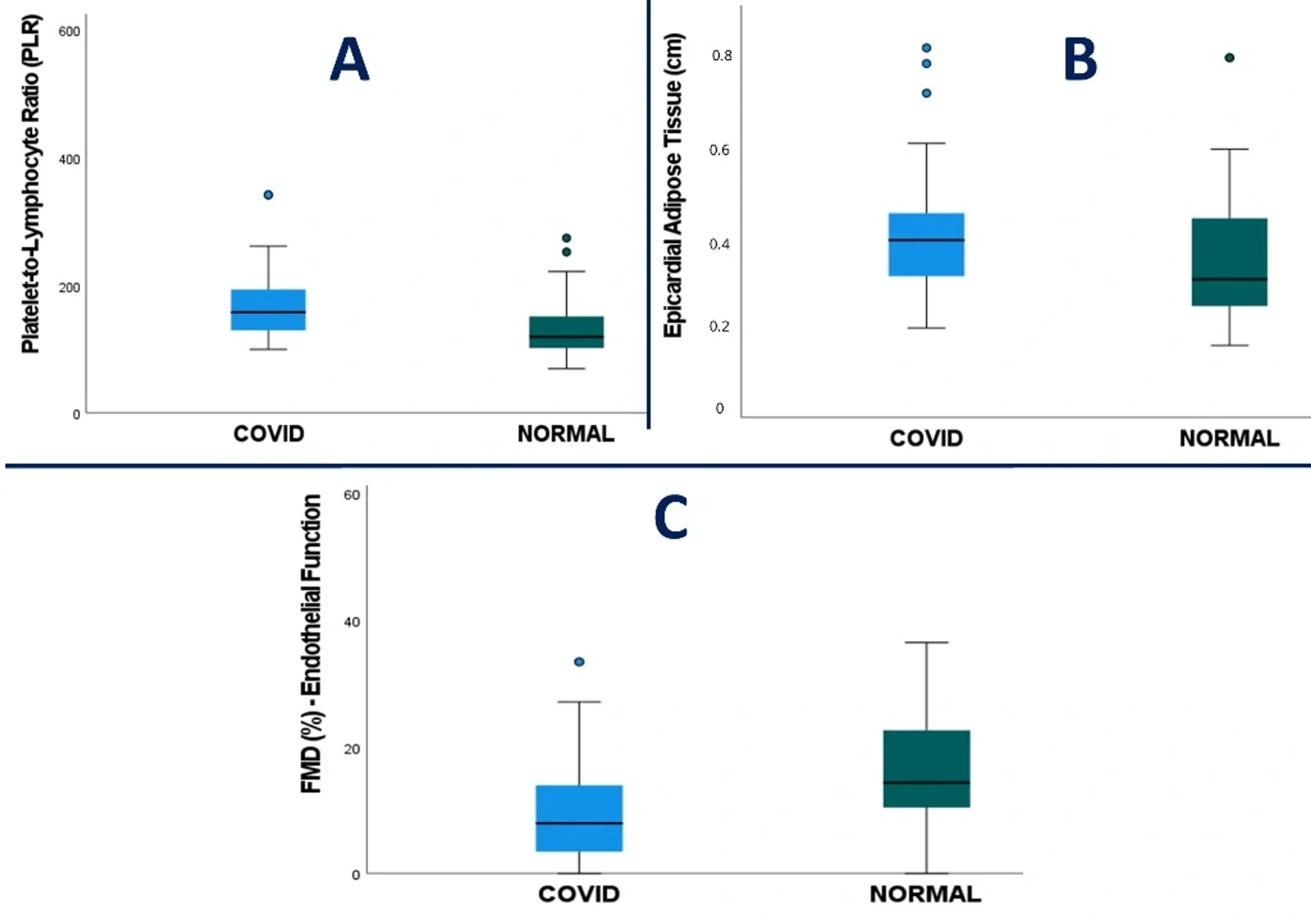
Box plot comparisons of platelet-to-lymphocyte ratio (A), epicardial adipose tissue (B), and flow-mediated dilation (C) between the COVID-19 and control groups This figure was created using IBM SPSS Statistics version 27.0 (IBM Corp., Armonk, NY, USA). FMD, flow-mediated dilation

Echocardiographic and ultrasonographic findings

Echocardiographic and ultrasonographic evaluations of the COVID-19 group were performed 43 days (range: 32-174; IQR: 43.7) after diagnosis. FMD was lower in the COVID-19 group (7.90 (3.45-14.09)% vs. 14.30 (10.32-22.56)%, p=0.005). EAT was thicker in patients with COVID-19 (p=0.021). Systolic pulmonary artery pressure (sPAP) and left ventricular diastolic diameter (LVDD) were lower in the COVID-19 group (p=0.013 and p=0.039). The remaining measurements were similar between the groups (Table 3, Figure 2).

**Table 3:** Echocardiographic and ultrasonographic measurements of the study population Data are expressed as mean±SD for normally distributed variables (*), and as median (25th-75th percentiles) for non-normally distributed variables (**). Note: Septal-lateral and lateral RV are presented as median (IQR) due to data distribution. *Independent samples t-test; *Mann-Whitney U test. EAT, epicardial adipose tissue; LVDD, left ventricular diastolic diameter; LVSD, left ventricular systolic diameter; EF, ejection fraction; IVCT, isovolumic contraction time; IVRT, isovolumic relaxation time; TAPSE, tricuspid annular plane systolic excursion; sPAB, pulmonary artery systolic pressure; FMD, flow-mediated dilation; LA, left atrium; RV, right ventricle

Variables	N (control/COVID-19)	Control group	COVID-19 roup	p-value
Aortic systolic diameter (cm)	44/36	2.34 (2.28-2.61)	2.50 (2.27-2.72)	0.270**
Aortic diastolic diameter (cm)	44/36	2.18±0.28	2.31±0.34	0.064*
Aortic diameter change (cm)	44/36	0.22 (0.16-0.31)	0.22 (0.13-0.39)	0.724**
Aortic strain	44/36	0.09 (0.07-0.14)	0.10 (0.06-0.14)	0.565**
Distensibility (kPa^-1^)	44/36	0.005 (0.004-0.008)	0.006 (0.003-0.008)	0.985**
Aortic stiffness index (%)	44/36	16.18 (11.44-21.07)	14.53 (11.36-24.81)	0.636**
EAT (cm)	44/36	0.31 (0.25-0.45)	0.40 (0.31-0.46)	0.021**
Left atrial diameter (cm)	44/36	3.01 (2.76-3.18)	3.03 (2.74-3.30)	0.828**
LVDD (cm)	44/36	4.51±0.47	4.30±0.43	0.039*
LVSD (cm)	44/36	2.80±0.36	2.67±0.36	0.111*
EF (%)	44/36	67.56±5.76	66.36±3.68	0.284*
IVCT (ms)	44/36	62.00 (53.00-67.00)	63.00 (53.75-76.25)	0.254**
IVRT (ms)	44/36	56.50 (53.00-63.00)	63.00 (56.00-69.25)	0.074**
S	44/36	14.60 (13.45-16.68)	14.90 (12.15-16.30)	0.753**
TAPSE (cm)	44/36	2.21 (2.02-2.64)	2.18 (1.89-2.45)	0.403**
sPAB (mmHg)	12/15	24.92±5.48	20.33±3.37	0.013*
FMD (%)	44/36	14.30 (10.32-22.56)	7.90 (3.45-14.09)	0.005**
LA maximum area (cm²)	44/36	17.75±2.61	16.85±3.17	0.169*
LA minimum area (cm²)	44/36	10.65 (9.49-12.30)	10.00 (9.10-12.55)	0.225**
Septal-lateral	44/36	14.00 (4.75-21.75)	19.00 (10.75-26.00)	0.066**
Lateral RV	44/36	15.50 (7.00-25.00)	21.00 (8.00-31.75)	0.215**

Rhythm Holter findings

Holter results of the study population are summarized in Table 4. There were no significant differences in HRV parameters between the control group and patients with COVID-19.

**Table 4:** 24-hour electrocardiographic Holter measurements of the study population Data are expressed as mean±SD for normally distributed variables (*), and as median (25th-75th percentiles) for non-normally distributed variables (**). *Independent samples t-test; *Mann-Whitney U test. rMSSD, root mean square of successive differences between adjacent normal-to-normal intervals; pNN50, proportion of NN50 divided by the total number of NN intervals; SDANN, standard deviation of the mean of all normal-to-normal intervals in all consecutive 5-min segments of the entire recording; SDNN, standard deviation of all normal-to-normal intervals; bpm, beats per minute

Variables	N (control/COVID-19)	Control group	COVID-19 group	p-value
RRmean (ms)	44/36	654.00 (618.00-707.25)	667.00 (617.50-699.25)	0.965**
RRSD (ms)	44/36	97.00 (89.00-124.75)	102.00 (86.75-129.25)	0.474**
rMSSD (ms)	44/36	23.00 (19.00-35.00)	24.00 (20.00-39.75)	0.663**
pNN50 (%)	44/36	4.00 (2.00-9.75)	4.50 (2.00-11.00)	0.665**
SDANN (ms)	44/36	84.00 (77.00-112.50)	87.00 (77.50-137.50)	0.276**
SDNN index (ms)	44/36	59.00 (51.50-63.75)	56.50 (49.50-72.25)	0.938**
Minimum heart rate (bpm)	44/36	63.52±10.73	64.89±12.75	0.604*
Mean heart rate (bpm)	44/36	90.75±7.89	91.47±7.75	0.682*
Maximum heart rate (bpm)	44/36	141.16±12.80	141.53±15.28	0.907*

Correlation and ROC analysis

Correlation analysis was performed for the total study population. FMD showed a negative correlation with PLR (r=-0.284, p=0.011) and pregnancy week (r=-0.230, p=0.040). FMD was positively correlated with LVDD (r=0.250, p=0.025). In subgroup analysis, epicardial fat thickness (EAT) was positively correlated with LVDD only in the COVID-19 group (r=0.471, p=0.004). No other significant correlations were observed. Furthermore, in the ROC curve analysis, PLR, FMD, and EAT discriminated a history of mild COVID-19 during pregnancy. PLR yielded an area under the curve (AUC) of 0.759 (95% CI: 0.656-0.863, p<0.001) with a cutoff value of >132.5 (sensitivity: 80.6%, specificity: 68.2%). FMD had an AUC of 0.682 (95% CI: 0.561-0.803, p=0.005) with a cutoff value of <11.2% (sensitivity: 69.4%, specificity: 75.0%). Finally, EAT showed an AUC of 0.651 (95% CI: 0.528-0.773, p=0.021) with a cutoff value of >0.35 cm (sensitivity: 72.2%, specificity: 61.4%) (Figure 3).

**Figure 3:**
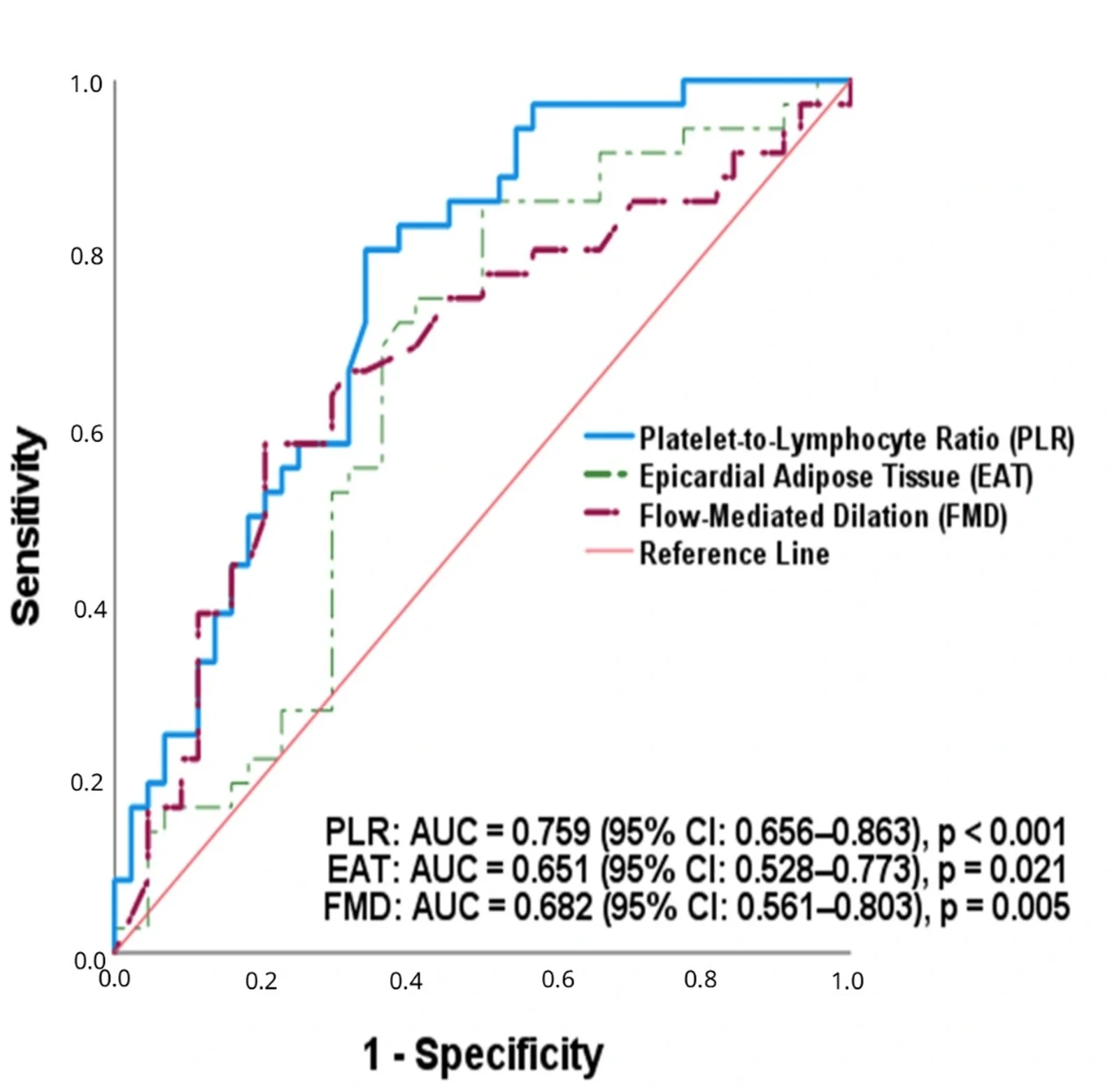
ROC curves of PLR, EAT, and FMD for predicting a history of mild COVID-19 during pregnancy This figure was created using IBM SPSS Statistics version 27.0. (IBM Corp., Armonk, NY, USA). ROC, receiver operating characteristic; PLR, platelet-to-lymphocyte ratio; EAT, epicardial adipose tissue; FMD, flow-mediated dilation

Regression analysis

Two multivariable linear regression models were evaluated to determine the independent predictors of FMD. In the first model, which included COVID-19 history and pregnancy week, adjusting for pregnancy week attenuated the association between COVID-19 history and FMD (unstandardized B=4.669, standardized beta=0.174, 95% CI: -1.281 to 10.619, p=0.122). The second model included pregnancy week, EAT, and PLR to predict FMD. In this model, advanced pregnancy week showed a negative association with FMD (unstandardized B=-0.449, standardized beta=-0.275, 95% CI: -0.807 to -0.090, p=0.015). However, PLR (unstandardized B=-0.032, standardized beta=-0.185, 95% CI: -0.069 to 0.006, p=0.097) and EAT (unstandardized B=-2.209, standardized beta=-0.025, 95% CI: -21.498 to 17.080, p=0.820) did not reach statistical significance.

## Discussion

In this study, we investigated the persistent cardiovascular and autonomic effects of mild COVID-19 during pregnancy. To our knowledge, this is the first study to demonstrate subclinical vascular endothelial dysfunction, evidenced by impaired FMD, in pregnant women recovering from mild COVID-19. These patients exhibited increased EAT thickness and higher PLR. Holter monitoring showed no autonomic differences (Figure 1).

As a result of physiological and immune functional changes in pregnant women, the risk of both contracting viral infections and disease severity increases [19,20]. In this regard, the Brazilian Ministry of Health included pregnant, postpartum, and post-abortion women in the high-risk group for COVID-19 [21].

Some of the changes that develop in the immune system during pregnancy are a shift in the CD4+ T cell population toward the Th2 phenotype instead of Th1 [22], a decrease in circulating natural killer (NK) cells [23] and plasmacytoid dendritic cells [23], and an increase in the level of progesterone, which is also a steroid hormone with immunomodulatory properties [24].

Nonetheless, while the evidence remains inadequate, COVID-19 in pregnancy seems to be less severe than infections caused by the H1N1 influenza virus, severe acute respiratory syndrome coronavirus (SARS-CoV), and Middle East respiratory syndrome coronavirus (MERS-CoV) [25]. Again, there is no evidence that COVID-19 causes spontaneous abortion, an increase in spontaneous preterm birth, or vertical transmission of SARS-CoV-2 [25].

Endothelial cells are essential in COVID-19 and many disease processes. The impact of the increase in blood volume, heart rate, stroke volume, and cardiac output, as well as the decrease in vascular resistance [26], on endothelial cell function in pregnant women is still uncertain. Numerous changes in systemic vascular physiology occur during adaptation to pregnancy. Endothelial damage and dysfunction in COVID-19 may result from direct infection of the endothelium by SARS-CoV-2 or an uncontrolled systemic inflammatory response [27].

FMD, a noninvasive ultrasonographic method, is widely used to evaluate endothelial function [28].

Holter analysis is a widely used method for evaluating many arrhythmias and HRV. A decrease in HRV reflects the cardiac effects of the autonomic nervous system and is considered a sign of autonomic dysfunction [29]. In COVID-19 infection, the vagus nerve is an essential modulator of the inflammatory pathway. Inflammation is inhibited as a result of the vagus nerve signaling the brain about peripheral inflammation [30]. Low vagal activity is reflected clinically as a cytokine storm [31]. Vagus nerve activity can be evaluated noninvasively with HRV [32]. In studies conducted on patients with COVID-19, the relationship between HRV and hospital stay, mortality, and ICU requirements has been studied [32,33].

Similar to our finding in pregnant women, Yi-Ping Gao et al. showed that persistent endothelial dysfunction on the 327th day after recovery (IQR 318-337 days) was associated with decreased FMD in a study comparing 86 participants who had COVID-19 with 28 healthy controls and 30 risk factor-matched controls [34].

The study conducted by Ergul et al., which included 63 patients with COVID-19 two months after recovery, identified COVID-19 infection and elevated BMI as independent predictors of endothelial dysfunction as assessed by FMD [35].

In another study, Riou et al. showed a relationship between disease severity and decreased FMD in 16 mild-moderate and nine severe-critical patients three months after disease onset [36].

The relationship between autonomic changes and disease findings in nonpregnant patients with COVID-19 has been studied in the literature using HRV [32,36,37]. Autonomic dysfunction, which may develop due to the underlying inflammatory process or treatment side effects, was not detected in our study of pregnant patients with COVID-19. Including patients with mild disease who would not require hospitalization in our study and treating them with supportive measures rather than COVID-19-specific medical treatment due to their pregnancy may have contributed to this different result.

We observed elevated PLR and reduced unadjusted FMD in the COVID-19 cohort. Laboratory samples were obtained at different times. COVID-19 samples were obtained during the acute phase, whereas control samples were obtained concurrently with cardiovascular testing. This limits the interpretation of inflammatory markers. Unadjusted FMD showed a negative correlation with PLR. This correlation disappeared within the COVID-19 group alone. This is a cross-sectional study, and we cannot establish causation. The elevated PLR and reduced FMD represent associations.

Pregnancy physiology induces vascular changes. Regression models showed an association between advanced pregnancy week and reduced FMD. PLR had no statistically significant effect on FMD in the multivariable model. The structural vascular changes of normal pregnancy complicate the evaluation of postviral effects. Future cohort designs require strict gestational age matching.

EAT is a highly inflammatory depot located between the myocardium and the visceral pericardium. EAT can be measured with both thoracic CT scans and transthoracic echocardiography (TTE). Hélène Bihan and colleagues analyzed the relationship between EAT volume measured by chest CT and clinicobiological and radiological parameters that may be related to the severity of COVID-19. They concluded that measuring EAT volume may help determine the prognosis of COVID-19 [38]. EAT measurement with TTE is especially important during pregnancy because it does not involve radiation. In our study, we found that EAT thickness in pregnant women with COVID-19 may be an indirect indicator of cardiometabolic inflammation in this patient group. Previous studies have highlighted the utility of noninvasive echocardiographic parameters in assessing subclinical myocardial and vascular changes in systemic inflammatory diseases, such as Behçet's disease [39]. In a similar context, our findings suggest that these noninvasive imaging methods may be useful in evaluating the potential subclinical vascular effects of COVID-19 in pregnant women.

The COVID-19 group showed lower sPAP. This contrasts with some reports of prolonged elevation [40]. White blood cell (WBC) and neutrophil counts were lower. Platelet counts and ALT levels were higher. These differences may reflect a postviral recovery state, mild liver damage, or normal pregnancy variations [41,42]. We found higher PLR in the COVID-19 group. The clinical relevance requires confirmation. Pregnant women with a history of COVID-19 may benefit from cardiovascular follow-up.

Study limitations

This study has major limitations. It is a single-center, cross-sectional study with a small sample size. The population consists of mild COVID-19 cases. The results may not generalize to patients with severe disease. We lack pre-infection baseline measurements and long-term follow-up records. Lower FMD or greater EAT thickness could have preceded the infection. Laboratory samples were collected at different times. Substantial missing data exist for parameters such as sPAP and D-dimer. For example, sPAP measurement requires a tricuspid regurgitation jet. This technical limitation makes the sPAP findings uncertain. The COVID-19 group had a more advanced pregnancy week. Normal pregnancy alters endothelial function. Adjustment for gestational age attenuated the main FMD findings. We did not control for unmeasured variables such as vaccination status. These exploratory findings require confirmation in age-matched longitudinal cohorts.

## Conclusions

There are still gaps regarding the course of COVID-19 in pregnant women. Our study showed lower unadjusted FMD and thicker EAT in subjects recovering from mild COVID-19. Autonomic function remained unaffected. Multivariable analysis did not confirm an independent association between COVID-19 history and FMD after adjusting for gestational age. The vascular changes of normal pregnancy act as a critical confounder. Evaluating these parameters in pregnant patients provides meaningful clinical insight despite these limitations. The long-term cardiovascular effects of the disease require clarification. These exploratory findings require confirmation in robust, age-matched longitudinal cohorts. Until then, closer cardio-obstetric surveillance to monitor subclinical alterations remains a hypothesis-generating concept.
